# Towards personalised follow-up care in ovarian cancer using online remote PROMs monitoring: a study protocol of a feasibility trial

**DOI:** 10.1136/bmjopen-2025-113371

**Published:** 2026-03-18

**Authors:** Johanna W M Aarts, S J Oudbier, M M Van Muilekom, P Röling, J Tromp, Marian Smeulers, Luc RCW van Lonkhuijzen

**Affiliations:** 1 Amsterdam UMC Location VUmc, Amsterdam, Netherlands; 2 Treatment Quality of Life, Cancer Centre Amsterdam, Amsterdam, Netherlands; 3 Department of Medical Psychology, Amsterdam UMC Location AMC, Amsterdam, Netherlands; 4 Digital Health, Amsterdam Public Health Research Institute, Amsterdam, Netherlands; 5 Division of Outpatient Department, Amsterdam UMC Location AMC, Amsterdam, Netherlands; 6 Child and Adolescent Psychiatry & Psychosocial Care, Amsterdam UMC Location AMC, Amsterdam, Netherlands; 7 Amsterdam Public Health, University of Amsterdam, Amsterdam, Netherlands; 8 Department of Obstetrics and Gynaecology, Amsterdam UMC Location VUmc, Amsterdam, Netherlands; 9 Department of Medical Oncology, Amsterdam UMC Location VUmc, Amsterdam, Netherlands; 10 Obstetrics and Gynaecology, Amsterdam UMC - Location AMC, Amsterdam, North Holland, Netherlands

**Keywords:** Quality of Life, Patient Reported Outcome Measures, Gynaecological oncology, Feasibility Studies

## Abstract

**Abstract:**

**Introduction:**

Ovarian cancer patients often experience persistent symptoms such as fatigue and pain, impacting their quality of life. Current follow-up care, focused primarily on recurrence detection, may not adequately address these symptoms and can be burdensome. This study evaluates the feasibility of remote monitoring using patient-reported outcome measures (PROMs) and measurements of weight and abdominal circumference as an alternative to standard hospital visits. We aim to assess feasibility (ie, usability and satisfaction) of this approach and identify implementation barriers and facilitators.

**Methods and analysis:**

This study is a single-centre longitudinal observational pilot that uses both qualitative and quantitative data to evaluate the feasibility of an innovative remote monitoring system for ovarian cancer follow-up care (Controle op Afstand, CopA). It is accessible to both healthcare professionals in the electronic health record and patients through the patient portal. Instead of 3-monthly in-hospital visits, patients are invited to complete regular surveys assessing PROMS about symptoms and quality of life and home measurements of weight and abdominal circumference. Feasibility will be assessed by (1) analysing patient and healthcare professional (HCP) experiences with CopA with the Measurement Instrument for Determinants of Innovations questionnaire for HCPs, and the ‘Experienced Usability and Satisfaction with Self-monitoring in the Home Setting’ questionnaire for patients, (2) investigating implementation barriers and facilitators using qualitative method and (3) performing a process evaluation of the intervention, assessing components such as reach, fidelity and compliance, time to response and number of (tele)consultations during the study period. Quantitative data will be analysed using descriptive statistics. Qualitative data will be analysed using thematic analysis.

**Ethics and dissemination:**

This study was reviewed by the Ethics Committee of the Amsterdam University Medical Centre (METC 2022.0256) and exempted it from further review as this study was not subject to the Dutch Medical Research Involving Human Subject Act. Results will be disseminated via peer-reviewed open-access publications, scientific conferences and targeted communication to patient organisations, healthcare providers and the wider public.

**STRENGTHS AND LIMITATIONS OF THIS STUDY**

- The intervention was rigorously developed in co-creation with multiple stakeholders, including different types of clinicians, patient representatives, members of the hospital-wide programme team for realising remote monitoring of patients, electronic health record specialists and a patient-reported outcome measures (PROM) implementation specialist from the PROM Expertise Centre.
- In this study, we will use a mixed methods approach, which enables a thorough understanding of the barriers and facilitators to the implementation of remote monitoring in follow-up care for ovarian cancer patients.
- The intervention will be implemented in daily clinical practice, which will provide valuable insights into real-life barriers and facilitators.
- As this is a feasibility study, it will not be able to draw conclusions on the effect of the intervention on health outcomes of patients.
- This is a single centre study in the Netherlands, which may limit generalisability to other countries.

## Introduction

 People diagnosed with gynaecological cancer are usually followed for a long time after their primary treatment. Each year, around 1300 women in the Netherlands are diagnosed with ovarian cancer.^1^ Most women are diagnosed with advanced disease and, despite surgery and chemotherapy, more than 75% will recur within 24 months. The diagnosis of ovarian cancer and its treatment can lead to (late) side-effects and affect diverse aspects of quality of life, such as physical, psychological and social problems. This can last for many years post-treatment and often never completely resolve.^2^

Current follow-up (FU) care in ovarian cancer in the Netherlands is mostly doctor-driven and includes a standardised 3-monthly schedule of hospital visits. This accounts for many European countries. The efficacy of this approach is, however, a subject of debate. Follow-up consultations primarily focus on detecting signs of recurrence, while there is also a need for addressing late treatment side effects and symptom management. Physical examination, which is often performed in FU consultation, is not well suited for its intended purpose of detecting recurrence.^3^ Furthermore, early detection of recurrence and prompt initiation of treatment in asymptomatic patients has no effect on survival rates or health-related quality of life (HRQoL).^4^ Early initiation of treatment could even pose a risk to HRQoL, due to significant treatment side effects.^4 5^ Therefore, standard imaging or monitoring of the CA-125 tumour marker is not deemed useful.^4^

To accommodate the individual needs and preferences of patients, oncologists need to have a holistic view of their situation and have a clear understanding of these specific needs. Women with ovarian cancer feel insufficiently involved in FU care and decision-making, with many needs unmet.^6 7^ This is partly due to the fact that the oncologist’s interpretation of the patient’s situation often does not align with the problems patients report themselves.^6^ Patients also struggle to determine where to seek assistance and guidance when encountering problems.^7^ Altogether, this illustrates the need for a personalised and tailored approach in FU ovarian cancer care.

The use of patient-reported outcome measures (PROMs) can help personalise FU care in patients with ovarian cancer. PROMs are standardised questionnaires that evaluate the patient’s perception of their own health status or well-being. When used in clinical practice, problems identified with PROMs can be acted on by the healthcare professional (HCP).^8^ Most studies used PROMs during active treatment or prior clinical consultations and can function as a dialogue tool to facilitate the exchange of all relevant information.^9^,^13^ It showed that this may lead to earlier recognition of symptoms, better symptom control, an increase in self-management and an increase in patients’ satisfaction and shared decision-making between patients and their HCPs. Using PROMs may even lead to improved survival.^11^ This provides opportunities for women in follow-up after ovarian cancer treatment. PROMs can help tailor FU care to the patient’s situation. Using remote Patient Reported Outcome (PRO) monitoring can be used to decide whether a patient needs an outpatient visit, possibly leading to better symptom assessment and more patient-centred care than current usual follow-up care in ovarian cancer patients. It can also potentially reduce the need for in-person hospital visits, as patients’ needs may be addressed through remote consultations. There are only a few examples where PRO-based follow-up is used instead of scheduled visits and as the basis for contact.^14^ The Ambuflex system, a generic PRO system used in Denmark, showed, for example, the impact on the organisation of care: almost half of epilepsy patients did not need further contact with the clinic after completing PROMs.^15^ A similar pilot study in breast cancer also showed that personalising FU care using electronic assessment of PROMs was cost-effective without diminishing patient’s satisfaction.^16^

There are two other studies, one in Denmark and one in the UK, that examined the use of PROMs with remote monitoring as a way to tailor follow-up care for ovarian cancer patients.^17 18^ Patients in these studies were overall positive, but the studies also state that more work and insight is needed to improve implementation of remote monitoring in the future.

This study aims to evaluate the feasibility of remote monitoring using PROMs combined with home measurement of weight and waist circumference as an alternative to regular hospital visits for follow-up in patients with ovarian cancer. We will assess the feasibility of the intervention by evaluating patients’ and healthcare providers’ experiences, conducting a process evaluation and identifying facilitators and barriers for implementation.

## Methods and analysis

### Setting

In the Netherlands, care for women with ovarian cancer is organised in regions around the nine gynaecological oncology centres. All patients are referred to one of these centres for their surgery while chemotherapy is mostly provided in the referring hospitals. National guidelines for ovarian cancer FU in the Netherlands allow for personalization of FU care. Patients may choose 3-monthly in-person hospital consultations with their gynaecological oncologist or medical oncologist, 3-monthly phone/email consultations, or they may choose to only contact their HCP themselves when they feel the need arises. Although national guidelines state that patients can choose between these different options, in practice this choice is rarely offered. As a result, FU care in our region mostly consists of 3-monthly in-person consultations. Our region covers approximately 20% of Netherlands gynaecological oncological care. In this setting, we will pilot test CopA (‘Controle op Afstand’, ie, Dutch for remote follow-up). This is an innovative approach to follow-up monitoring of symptoms and quality of life aspects remotely, for patients who finished primary treatment for ovarian cancer.

### Intervention

#### Development of the intervention (CopA)

CopA consists of a technical application and of a new process to review and act on the data generated by patients. CopA was developed using a structured 3-step approach. We started with an intake phase (step 1) with the clinical project lead of the department to discuss the potential goals, map the current process and draft the desired process in concept. To create support and jointly design the new process, a multidisciplinary stakeholder group was formed, including former patients with ovarian cancer, representatives of the healthcare providers involved in follow-up care, that is, clinical project lead, gynaecological oncologists, medical oncologists, physician assistants and oncological nurses. In addition, members of the hospital-wide programme team for realising remote monitoring of patients were involved, that is, programme leader, project leader, electronic health record (EHR) specialists, PROM implementation specialist from the PROM Expertise Center^19^ and a researcher. The second step was the design phase in which we discussed the potential goals and impact of CopA and the concept process for remote monitoring with the multidisciplinary stakeholder group in a focus group setting. This resulted in consensus on added value of CopA, shared goals and tuning of the desired process, including eligibility criteria for participation in remote monitoring, measurements and frequency of collecting measurements, process for monitoring, communication preferences and escalation procedures. The participation of patient representatives in this process was invaluable. They provided input on the need for a defined starting point of the follow-up phase, a clear explanation on added value and safety of remote monitoring compared with the current follow-up care, expectations regarding self-management and the need for reminders and preferences on feedback and contact. In the third step (building phase), we developed the remote monitoring application within the EHR, established work processes in a protocol and developed patient information (website and manuals). During this phase, issues were regularly discussed with the core project team, and the final application and work processes were presented and discussed with the multidisciplinary stakeholder group, resulting in a finalised product for use in the study.

The TiDIER-telehealth checklist was used to systematically describe all elements of CopA (online supplemental file I).^20^

#### Components of CopA

##### Measurements

The stakeholder group reached consensus on the integration of the following PROs and measurements to be monitored. These questionnaires are specifically targeted to measure symptomatic side effects in oncology and determine HrQoL of cancer patients. In table 1­, a full list with details of these PROMs is included. The stakeholder group defined thresholds and corresponding action for each measurement which can be found in the same table.

**Table 1:** Predefined thresholds triggering an alert for each PROM and measurement

Name of PROM	Thresholds		Interpretation
EORTC QLQ C30*	See figure 1 for thresholds for clinical importance		Scores in orange will send an alert to the daily report visible to oncological nurse. These scores are also visible to the patient.
EORTC – OV 28	There is no existing literature on thresholds for this questionnaire		No alert system created
LARS questionnaire†	**Score**	**Interpretation**	
	0 – 20	No problems, no LARS	No alert
	21 – 29	Number of problems, low LARS	Yes, alert
	30 – 42	Many problems, high LARS	Yes, alert
SNAQ‡	**Score**	**Interpretation**	
	≥ 2	Moderately malnourished	No, alert
	≥ 3	Severe malnourished	Yes, alert
Weight	A weight change of 10% (both increase and decrease) triggers an alert		
Abdominal circumference	No predetermined threshold.		

* Based on Giesinger et al. 2020

† Based on Hupkens et al. 2018.

‡ Based on Kruizenga et al. 2005.

LARS, low anterior resection syndrome; PROM, patient-reported outcome measure.

**Figure 1:**
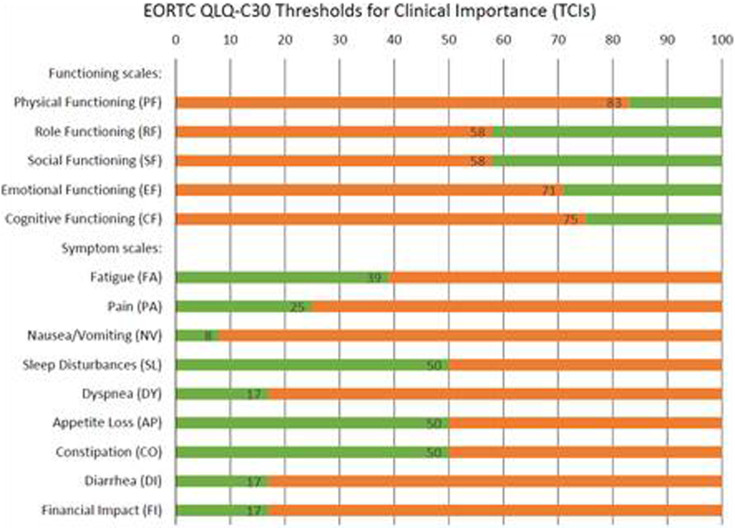
EORTC QLQ C-30 Thresholds for Clinical Importance used a to alert. Based on Giesinger, JM, *et al.*

The following PROMs are used:

- The EORTC Core Quality of Life Questionnaire (EORTC-QLQ-C30).^21^
- The additional module for ovarian cancer patients (EORTC – OV28).
- The short nutritional assessment questionnaire (SNAQ).^22^
- The low anterior resection syndrome (LARS) questionnaire, in case patients underwent a low anterior resection during debulking surgery.^23^

The following measurements are added:

- Body weight (in kg).
- Abdominal circumference (in cm).

These measurements were added after discussion in the multidisciplinary stakeholder group. Particularly, the patient representatives were in favour of adding these. Patients had experienced an increase in abdominal circumference or weight change at the time of diagnosis. Adding these measurements would increase the feeling of self-management and control of patients.

Additionally, open text fields are available where patients can report the presence and severity of any other symptom. For safety reasons, patients will be instructed to call the hospital immediately in case of an emergency (the usual instructions, such as vomiting or abdominal pain) and to contact the HCP if they have any doubts about their symptoms. To increase personalization and self-management of FU care, patients will always have the option to deny contact even if their results trigger an alert. Alternatively, patients can always request contact even if no threshold is exceeded. Both options will allow patients to elaborate on their decision in an open text field.

##### Procedures and work flow

- CopA is initiated with a designated hospital visit at the end of treatment. During this visit, explanation and information are provided about CopA. An instruction video is available on how to perform the home measurements.
- Three-monthly, patients receive an automatic invitation to complete the PROMs in the patient portal (MyChart) of the EHR (table 1) and to measure and provide their weight and abdominal circumference. The invitation for completion is sent 14 days before the deadline, giving patients the freedom to complete the survey in their own time. Completion is encouraged through reminders sent through email/online application 1 week before the deadline. Patients will answer the questionnaire based on their perception of their current well-being. Patients can access the patient portal and home monitoring system at any time.
- On the hospital side, a daily report is created of the patients that have completed a new series of PROMs. It allows the oncological nurse to evaluate the responses to the PROMs and take appropriate actions if indicated. Table 2 shows the type of action required for the different types of alerts. An alert is generated in the EHR, visible to the oncological nurse when the predefined thresholds are exceeded, indicating a need for intervention. An active approach is used wherein the HCP is instructed to contact the patient within 48 hours. The nurse specialist will contact the patient to together decide on the best course of action (ie, visit to the hospital, referral to other HCPs or watchful waiting). The patient also has the option—at the end of the questionnaire—to state that contact from the hospital is not needed or preferred. Patient representatives requested this option, which is in line with patient preferences in another comparable study among lung cancer patients that strongly preferred this option to be added.^10^

**Table 2:** Possible actions based on home measurement outcomes

No threshold(s) exceeded and no specific wish for contact*	The nurse sends a message to the patient that no further action is needed and that values and answers to PROMs look good.
No threshold(s) exceeded, but explicit wish for contact	(Nurse will discuss with the gynaecologist or oncologist.) The nurse sends a message to the patient that the values and answers look good, but that the patient will be contacted for an appointment/ patient can make an appointment.
Threshold(s) exceeded, but wish for no contact	(Nurse will discuss with the gynaecologist or oncologist.) The nurse sends a message to the patient that some of the values or answers exceed the threshold, and that this might be a reason for contact with the nurse or doctor. However, as the patient indicates no need for contact, an appointment will not be made. The patient will be informed that they can always seek contact if they change their mind based on this information.
Threshold(s) exceeded and open for contact	(Nurse will discuss with the gynaecologist or oncologist.) = Thresholds exceeded⇒patient is contacted by nurse by telephone for further action.
Patient failed to complete PROMs	An alert will be sent to the oncological nurse. The patient receives another online reminder. When there is no response, the nurse will call the patient after 1 week at the latest.

* Patients will have the option to click ‘no contact wanted’ which may be used when the patient prefers self-management or if symptoms reflected in the PROMs may have an external cause; eg, mood.

PROM, patient-reported outcome measure.

- Based on the outcomes of filled out PROMs and/or contact with the nurse, patients might need support from a HCP outside of the hospital, that is, physical therapist or dietician. In the patient portal, a link to a website with a comprehensive list of information is made available for patients helping them to find the appropriate HCP nearby their home. They can consult this list at any time in their portal facilitating self-management when patients notice a problem based on the PROMs they fill out, regardless of exceeding a threshold.
- Patients always have the option to call the hospital when experiencing problems.
- Patients will have one hospital visit after 6 months.

### Study design

This is an observational single centre pilot study using qualitative and quantitative data, aiming to assess feasibility of the CopA intervention. Data on feasibility and satisfaction with CopA will be collected from both HCPs and from patients accepting the intervention, through questionnaires and interviews. During the course of the study, a process evaluation will be performed to identify barriers and facilitators. We followed recommendations from the Strengthening the Reporting of Observational Studies in Epidemiology (STROBE) guidelines while reporting the study.^24^

**Figure 2:**
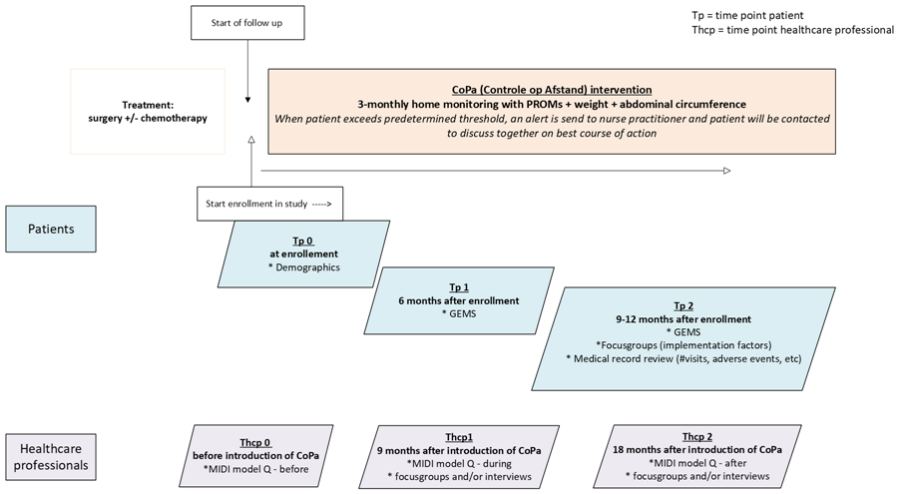
Study design.

The study design is depicted in figure 2.

### Patient population and in- and exclusion criteria for CopA

This study is aimed at all patients who received primary curative treatment for ovarian cancer (all FIGO (i.e. International Federation of Gynecology and Obstetrics) stages) and receive FU care. Patients will be included if they are within the first 2 years after the end of primary treatment. A small group of patients who receive maintenance therapy (with poly-ADP-ribose-polymerase inhibitors) will be excluded from this study, as they receive a different FU care plan with regular blood tests, scans and examinations at the outpatient department.

Patients are eligible for participation if they meet the following criteria: (1) completion of primary treatment for ovarian cancer less than 2 years ago, (2) having Internet access and sufficient degree of digital literacy as estimated by a HCP, (3) ≥18 years old and (4) ability to use the patient portal (MyChart). Patients are ineligible once they have a recurrence or if they lack sufficient proficiency in Dutch, since the patient portal is currently only available in Dutch.

### Patient recruitment for this pilot study

Patients are assessed for eligibility by their HCP involved with their treatment and FU care. Eligible patients who ended primary treatment will be given the choice to either receive this alternative form of FU care (ie, CopA) or proceed with the standard 3-monthly FU care, as currently employed in our region. Patients who are willing to participate will be informed about CopA by their HCP and case manager (ie, oncological nurse). They may always change their decision and return to standard 3 monthly FU care. Specific patient information (patient leaflets and short video clip) will be developed to support patients in decision-making. Informed consent will be obtained by their lead physician.

After obtaining informed consent, the case manager provides the participant with the instruction about the monitoring and a measuring tape to measure waist circumference at home. Patients are instructed to use their own weighing scale. Patients will receive a video and a website link including detailed information and explanation about CopA. The research team will be available to provide technical assistance for the duration of the study. Importantly, at the start of the study, it is emphasised that if patients need medical attention, they should contact the hospital by telephone at all times. Patients who experience a recurrence of disease during the study period will no longer participate in the home monitoring programme as they require a different type of care and treatment.

Recruitment started in November 2024 and to date 26 patients have been included in the study. Anticipated end date of the study is mid 2027.

### Data collection

For all patients background information is collected from the EHRs at T0. This includes age at inclusion, histological type and grading of ovarian cancer, FIGO stage, type of treatment, end date of treatment, smoking status and body mass index.

To evaluate the intervention, data will be collected to (1) analyse patient and HCP experiences with CopA, (2) investigate implementation barriers and facilitators using qualitative methods and (3) process evaluation of the intervention, assessing components such as reach, fidelity and compliance, time to response and number of (tele)consultations during the study period. The study coordinator will keep a logbook of all small adjustments that will be made during the implementation.

#### Patients and HCPs’ experiences with CopA

The experience of HCPs working with remote ambulatory monitoring of ovarian carcinoma, that is, oncological nurses, oncologists and gynaecologists, will be assessed using the Measurement Instrument for Determinants of Innovations (MIDI) questionnaire. This questionnaire is designed to evaluate the implementation of an innovation such as guidelines, protocols and digital technology.^25^ An adapted version of the MIDI will be distributed after the first moment of implementation (T0) to investigate HCPs’ *expectations* about working with remote monitoring and the possible barriers they think they might encounter.^24^ This adapted version includes only the relevant questions that HCPs are able to answer at the start of the project (expectations). After 6 (T1) and 12 (T2) months, the questionnaire will be sent again to assess actual experience. The MIDI questionnaire at T0, T1 and T2 will be used to determine if implementation barriers and facilitators change over time. The MIDI questionnaire covers four different domains, that is, the innovation itself (CopA), the user (HCP and patient), the organisation (the hospital) and the context (society).

Patients will receive the Experienced Usability and Satisfaction with self-monitoring in the home Setting (GEMS) questionnaire at T1 (6 months) and T2 (12 months). This validated questionnaire is designed to measure experienced usability and satisfaction of patients with self-management tools.^26^ The GEMS comprises four subscales: Convenience of use, Perceived value, Efficiency of use and Satisfaction. Each subscale consists of two items with a Likert scale from 1 to 5 (completely disagree to completely agree), except for the Satisfaction subscale, which consists of three items. Two of the items on the Satisfaction subscale have a Likert scale from 1 to 10.

Text box 1 provides a short overview of measurement instruments. In the online supplemental file II, we outlined the measurement instruments MIDI and GEMS in more detail, including response categories.

Box 1 **Short overview of questionnaires distributed at various moments in time to patients and HCPs who are involved in remote FU care for ovarian cancer**

*For healthcare professionals*

T-0hcp

- Baseline demographics: Age, gender, specialism in medicine, number of years working in the field.
- Adapted version of the MIDI questionnaire (*expectations*), including only relevant barriers/facilitator questions related to expectations of working with the technology.

Thcp-1 (after 6 months)

- Full MIDI questionnaire to identify barriers/facilitators based on experience with remote ambulatory monitoring.
- Short interviews with participating HCPs to clarify the identified barriers/facilitators including any problems they encounter when using remote monitoring, and to collect suggestions for improvement of ovarian cancer monitoring via the EHR/patient portal.

Thcp-2 (after 1 year)

- Full MIDI questionnaire to identify barriers/facilitators based on experience with the technology and to compare changes over time.

*For patients*

T-0p

- Baseline demographics: Age, gender, education level*, digital literacy**, health literacy**.
- QoL data from the EORTC QLQ C30 and OV-28.

Tp-1 (6 months after start of follow-up care)

- GEMS questionnaire: experienced usability and satisfaction after working with self-management via patient portal.
- Focus groups will be held with patients to clarify the results including any problems they have when using the system, and to collect suggestions for improvement of ovarian cancer monitoring via the EHR/patient portal.
- QoL data from the EORTC QLQ C30 and OV-28.

Tp-2 (1 year after follow-up care)

- GEMS questionnaire.
- QoL data from the EORTC QLQ C30 and OV-28.

EORTC Core Quality of Life Questionnaire (EORTC QL), HCP: Healthcare professional, Thcp:, MIDI: Measurement Instrument for Determinants of Innovations, OV-28: Ovary - 28, QoL: Quality of Life; GEMS: Experienced Usability and Satisfaction with self-monitoring in the home Setting.*Educational level is assessed according to the Dutch system, including categories no formal education; less than high school; high school diploma, some college, bachelor’s degree and graduate degree.**Digital literacy is assessed using one question as part of the validated GEMS questionnaire; health literacy is assessed as self-reported health literacy using the three validated questions by Chew et al. (2008).

Data collection of questionnaires will be done electronically by e-mail using Castor EDC. This is an electronic data capture platform designed to streamline the process of collecting, managing and analysing research data.

#### Identification of barriers and facilitators to implementation

Based on the results of the GEMS questionnaires and the MIDI questionnaires (see 1), in-depth interviews with HCPs and focus groups with patients will be performed to gain more insight into experiences, but also barriers and facilitators of the use and implementation of CopA in clinical practice. At the time of inclusion, patients are able to provide consent to be approached for additional focus groups. All patients who participate in CopA for at least 6 months and provide consent will be invited to a focus group (4–8 participants). For patients who relapsed, we will ask the treating physician if it is appropriate to approach the patient. We aim at organising two focus groups. All participating HCPs will be invited for a semi-structured interview after filling out the MIDI questionnaire at T1 (6 months). Both the focus groups and interviews will be conducted by researchers who are not involved in patient care and have experience in performing semi-structured interviews and leading focus groups. Both interviews and focus groups are audio-recorded and transcribed literally.

#### Process evaluation of CopA

For the process evaluation, we follow the model of Linnan and Steckler.^27^ This is a commonly used tool to evaluate the process of the implementation of eHealth interventions systematically because it describes the adherence to the intervention in five terms: (1) the proportion of intended target audience that participated in the study—reach, (2) the number of intended units of each component that was delivered—dose delivered, (3) the number of participants that actively engaged the delivered components of the intervention—dose received, (4) the extent of the intervention that was delivered as planned—fidelity and (5) participants’ satisfaction and usage barriers of the intervention—participants’ attitudes. Data for this process evaluation were collected from the patients using online questionnaires at T0, T1 and T2 and using qualitative methods as described above. In addition, data were also obtained from patients records and by means of an automatically generated weblog of the EHRs. Table 3 gives an overview of these process measures.

**Table 3:** Process measures, definitions and data collection methods

Process measure	
*Reach* Proportion of the target population that received the intervention	*Definition* Proportion of recruited potential participants that met all inclusion criteria and decided to engage in the study
	*Data collection method* Study database Weblog
*Dose delivered* Proportion of intended intervention that was actually delivered to target population	*Definition* Proportion of study population that received an account for the patient portal and first invitation to fill out PROMs
	*Data collection method* Weblog
*Dose received* Extent to which the participants used the intervention as recommended	*Definition* Proportion of patients with an account that used the patient portal to fill out PROMs and/or home measurements at least once
	*Data collection method* Weblog
*Fidelity* Extent to which the intervention was delivered as planned	*Definition* Proportion of patients who participated in the study for at least a year and received their 6 month appointment Time to response of the nurse to the measurements Number of (telephone) consultations during the study period
	*Data collection method* Weblog Patient records
*Participants’ attitudes* Satisfaction Perceived effectiveness Usage barriers Suggestions for improvement	*Target* Patients Gynaecologists and nurses
	*Data collection method* Interviews and focus groups MIDI questionnaire HCPs GEMS questionnaire patients

GEMS, the Experienced Usability and Satisfaction with self-monitoring in the home Setting questionnaire; HCP, healthcare professional; MIDI, Measurement Instrument for Determinants of Innovations; PROMs, patient-reported outcome measures; Weblog, data extracted from the EHR.

##### Reach

Reach concerns the degree to which an intended audience participated in the intervention. The research team identifies from the EHRs which patients finish treatment for ovarian cancer and enter follow-up. We register what proportion of these patients decides to participate in the study and who declines. Reasons for exclusion will be registered, as well as the number and reasons for drop-out during the study.

##### Dose delivered

Dose delivered refers to the proportion of the intended intervention that is actually delivered to the participants. The number of patients that received the online invitation after 3 months to fill out PROMs divided by the total number of participating/included patients was defined as dose delivered.

##### Dose received

Dose received is a measure of the extent to which participants actively engage with the intervention. Dose received was defined as the proportion of patients that used the intervention as recommended, that is, they filled out the PROMs and home measurements at least once. In addition, we monitor if patients had contact with the nurse practitioner either through an e-consult or telephone consultation.

##### Fidelity

Fidelity refers to the quality of the deliverance of an intervention and the extent to which the intervention was delivered as planned. We define fidelity in this study as the proportion of patients that filled out PROMs every 3 months for at least a year and received their 6-month appointment with the gynaecological oncologist. Furthermore, we will evaluate how often the answers of patients to the PROMs exceeded a threshold and what consequential actions were taken by the HCPs and/or patients. We will also determine the frequency and type of contact between the oncological nurse and the patient and the frequency of any additional appointments with a gynaecologist.

##### Participants’ attitudes

These will be assessed using the MIDI questionnaire and GEMS questionnaire (see under 1) and through interviews and focus groups (see under 2).

### Sample size

We aim to recruit 50 patients into the study in 2 years. This sample size is based on comparable feasibility studies.^28 29^

### Data analysis plan

#### Patients and HCPs’ experiences with CopA

Quantitative data collected through the MIDI and GEMS questionnaires will primarily be analysed using descriptive statistics. The MIDI results will be used to identify key implementation determinants, highlighting the most frequently cited factors that facilitate or hinder the implementation process. Mean scores per determinant will be calculated. A higher mean score indicates that an HCP perceives this determinant less as a barrier to implement CopA; higher scores are associated with higher expected levels of use.^30^ Differences between implementers and non-implementers will be assessed.

The GEMS questionnaire will provide insights into patients’ perceived usability and satisfaction with remote monitoring. In order to calculate the GEMS score, all items of the GEMS must be completed. In order to calculate the GEMS score, all items of the GEMS must be completed. To calculate participants’ satisfaction score, Likert scales of the GEMS questions will be revised from a 10-point to a 5-point scale, with values of 10 and 9 revised to 5, and 8 and 7 revised to 4, respectively, with higher scores indicating higher satisfaction. This adjustment was made for comparison of the items and to maintain consistency with the other items of the GEMS Satisfaction subscale. In order to calculate the subscale and overall GEMS scores, positively framed items from the GEMS were calculated in line with other usability metrics.^26^ Reversed items were calculated by subtracting the score from 5 (5-Score). Higher scores indicate better usability and satisfaction, with higher values reflecting more favourable perceptions of the system’s usability. The total GEMS score could reach a maximum score of 36 (100%) and was normalised by dividing the raw score by 36 and multiplying the score by 100.

#### Identification of barriers and facilitators to implementation

This study will generate qualitative data from interviews and focus groups with patient participants and HCPs at our clinic. These data will be analysed using reflexive thematic analysis based on the methods described in Braun and Clarke.^31^ Reflexive thematic analysis positions meaning as constructed through language and context. Two researchers (one junior and one senior researcher) will conduct line-by-line inductive, semantic coding using MAXQDA. Importantly, these researchers are not involved in ovarian cancer follow-up care and are not direct colleagues of the HCPs participating in this study, warranting an objective view. Both researchers independently perform this initial coding, whereafter they review the codes together and reach consensus on the final list of codes. Themes will be constructed to capture patterns of barriers and facilitators of the intervention in clinical practice on repeated, reflective engagement with the data. After these themes are developed, discussions with the broader research team, including two patient representatives, a clinician and implementation specialist, will take place contributing to critical interpretation of the constructed themes. The analysis and reporting of these findings will follow the Consolidated criteria for Reporting Qualitative Research.

#### Process evaluation of CopA

The quantitative data will be analysed using descriptive statistics such as calculation of proportion, frequencies, means and SD for all the aspects of the first four process measures.

### Patient and public involvement

Focus groups with patients with ovarian cancer^7^ and our patient sounding board taught us that the organisation of follow-up care needs change. Patients expressed a need for more self-management and more personalisation, leading to the idea for this project. During the development of CopA, two patient representatives were involved and repeatedly asked for feedback on the design of CopA as well as the implementation strategy in clinical care. They also reviewed the patient leaflets and online patient information to check that the language was suitable. A lay summary of our study findings will be developed and disseminated through Olijf, the patient organisation for women with gynaecological cancer.

### Ethics and dissemination

This study conforms to the Declaration of Helsinki. The medical ethical review board of Amsterdam University Medical Centres exempted this study from further assessment (METC 2022.0256) as this study was not subject to the Dutch Medical Research Involving Human Subject Act and approved the study protocol. In case of changes to the protocol during the study, these will be communicated to the ethics committee. The study is partially funded by a Clinical Fellow Grant from ZonMw (grant number 09032212110039; personal grant awarded to J.W.M. Aarts). The funder will not be involved in data analysis or report writing. Participant confidentiality will be ensured by anonymising outcome data by using identification numbers in data analysis. Interviews and focus group discussions will be led by researchers, not directly involved in care of participants. In Dutch national guidelines regarding follow-up care after ovarian cancer, it is stated that patients are allowed to choose their preferred route of FU, ranging from no regular appointments to phone consultations only to 3 monthly hospital visits. Studies have shown that omitting regular hospital visits does not affect oncological outcome.^29^ Therefore, there are no risks involved with regard to participation in this study. Nonetheless, each participant has the possibility to stop the study at any time and return to the standard follow-up protocol. At the end of the study period of 2 years, each participant will be asked to keep access to the home monitoring platform or, if they prefer, to return to standard care. The findings of this study will be published in peer-reviewed journals, presented at relevant conferences and disseminated to the public via newsletters of patient association for gynaecological cancer Olijf and through website and social media channels of ZonMw and Amsterdam University Medical Centers. All outputs will be authored and approved by the research team. Access to the full protocol including all measurement instruments and questionnaires used is available on request to the corresponding author. The principal investigator (ie, J.W.M. Aarts) declares no financial or competing interests.

## Discussion

This feasibility study will provide valuable insights into the implementation of remote monitoring using PROMs combined with home measurement of weight and waist circumference as an alternative to regular hospital visits for follow-up in patients with ovarian cancer. We will evaluate experienced usability and satisfaction from both the patients’ and HCPs’ perspectives, combining it with a process evaluation.

A strength of this study is that PROMs are already integrated into current workflows, so both patients and HCPs are familiar with the questionnaires and online patient portal. Another strength is the mixed-methods design in which we combine validated measurement instruments to assess feasibility and qualitative methods to gain more in-depth insight into their experiences. The process evaluation will guide further implementation efforts.

Some limitations should be mentioned as well. Although we do not select patients on having sufficient digital skills, the voluntary nature of participating in this study may introduce a selection bias, as it might scare patients who struggle with technical applications. Consequently, the findings may not fully represent the usability and satisfaction across the entire population. Another limitation of the study is the requirement for Dutch language proficiency, which may also exclude a portion of the patient population and affect the representativeness of the sample. Additionally, the fact that it is a single centre study may also negatively affect the generalisability of the results.

Future studies are needed to assess the actual impact of this form of follow-up care on patient outcomes, such as oncological outcomes, quality of life and costs.

In short, the findings of this study will provide valuable implementation lessons for remote monitoring in ovarian cancer follow-up care using PROMs, potentially offering patient-centred care to these women.

## Supplementary material

10.1136/bmjopen-2025-113371 online supplemental file 1

10.1136/bmjopen-2025-113371 online supplemental file 2
