# Supplementary material for: Towards personalised follow-up care in ovarian cancer using online remote PROMs monitoring: a study protocol of a feasibility trial
Source: BMJ Open. 2026 Mar 18;16(3):e113371. doi: 10.1136/bmjopen-2025-113371 (PMC13007198; doi:10.1136/bmjopen-2025-113371)
Supplement: online supplemental file 1 [file bmjopen-16-3-s001.docx]

**APPENDIX I. TIDIER Telehealth Checklist (Template for Intervention Description and Replication)**

| Item number | **Item** | **Description** | **Where located** |
| --- | --- | --- | --- |
| 1. | **BRIEF NAME** | Short name is ‘Controle op Afstand’ (i.e. remote follow up) | Under methods, page 5 |
| 2. | **WHY** | Current follow up care for ovarian cancer consists of 3-monthly visits to the hospital. However, it does not contribute to earlier detection of a recurrence while not accommodating to women’s needs. This intervention including PROMs home monitoring aims to tailor follow up care to the individual woman’s need. | Under introduction, page 4 |
|  | **WHAT** |  |  |
| 3. | Materials | Participants receive a patient leaflet including instructions. In their MyChart a link is included leading to information on the hospital’s website and some video clips with information | Under methods, page 10 |
| 4. | Procedures | Participants are supported by the oncological nurse which they can contact at any time by phone or through MyChart. There is also a digital support team at the hospital that could help solve technological issues participants experience during the study. Participants always receive a message from the nurse after they checked their answers. | Under methods, page 7 |
| 5. | **WHO PROVIDED** | The oncological nurses all received a 1-hour training on how to instruct patients and how to work with the technology. During the study the oncological nurses are supported with a member of the Home monitoring team from our hospital. | Under methods, page 7 |
| 6. | **HOW** | Patients will be invited to participate face-to-face by their healthcare provider. The oncological nurse will provide a short instruction face-to-face. The intervention is delivered through MyChart. They receive an alert by e-mail or text message on their phone when the PROMs and home measurements can be filled out. | Under methods, page 10 |
| 7. | **WHERE** | The intervention is integrated into EPIC, the hospital’s electronic health record. Patients can access it through MyChart, a platform to access their personal health record. | Page 7 |
| 8. | **WHEN AND HOW MUCH** | The patient will receive the invite to fill out PROMs and measurements every 3 months for a period of two years or until a recurrence of disease is detected. | Under methods, page 7 |
| 9. | **TAILORING** | This intervention aims at tailoring follow up care to the individual patient. Based on the patient’s responses to the home monitoring survey, a nurse will contact the patient and will decide together on the best next step depending on what the patient needs. | Under methods, page 7-9 |
| 10. | **MODIFICATIONS** | Not applicable. This is a study protocol. We plan to keep a log book of possible modifications on the intervention that will be done during the study. | Not applicable |
|  | **HOW WELL** |  |  |
| 11. | Planned | Adherence will be assessed. When a participant does not fill out the 3-monthly home monitoring survey, she will be contacted by our nurse. A process evaluation will be performed assessing data we can extract from the electronic health record to evaluate the participant’s use of the platform. | Under methods, page 9 |
| 12. | Actual | Not applicable. This is a study protocol | Not applicable |

Based on BMJ 2014; 348: g1687, and Rhon *et al.* 2022
