# Supplementary material for: Towards personalised follow-up care in ovarian cancer using online remote PROMs monitoring: a study protocol of a feasibility trial
Source: BMJ Open. 2026 Mar 18;16(3):e113371. doi: 10.1136/bmjopen-2025-113371 (PMC13007198; doi:10.1136/bmjopen-2025-113371)
Supplement: online supplemental file 2 [file bmjopen-16-3-s002.docx]

Supplementary file II **Measurement instruments used to evaluate feasibility of the intervention**

**____________________________________________________________________________**

**For healthcare professionals – Adjusted version of the** Measurement Instrument for Determinants of Innovations (MIDI)

Reference: Fleuren MA, Paulussen TG, Van Dommelen P, Van Buuren S. Towards a measurement instrument for determinants of innovations. Int J Qual Health Care. 2014;26(5):501-10.

**__________________________________________________________________________________**

**Innovation Technology**
The following questions concern your expectations regarding the implementation of home monitoring. In these questions, home monitoring refers to: remotely monitoring (using PROMs) the health status of patients with ovarian cancer during follow up.

For all items answering categories are:

- Strongly disagree
- Disagree
- Neither agree nor disagree
- Agree
- Strongly agree
- No opinion

3.1 I expect that home monitoring is clear: it is clear which activities I need to perform and in which order.

3.2 I expect that home monitoring is based on factually correct knowledge.

3.3 I expect that the materials and information needed to work properly with home monitoring are available.
3.4 I expect that home monitoring is complicated to use.
3.5 I expect that home monitoring fits well with how I am used to working.
3.6 I expect that the effects of home monitoring are clearly visible.
3.7 I expect that home monitoring is suitable for my patients.

**Healthcare Provider Perspective**
4.1 I expect that home monitoring helps me to identify the health problems of my patients.
4.2 I expect that home monitoring helps me in communication with the patient.
4.3 I expect that home monitoring will lead to more phone calls to the outpatient clinic.
4.4 I expect that home monitoring will lead to many Inbasket messages.
4.5 I expect that home monitoring helps with early identification of problems in the patient.
4.6 I expect that home monitoring gives my patient better insight into (the consequences of) her disease and treatment.
4.7 I consider it my responsibility as a healthcare provider to use home monitoring.
4.8 I expect that patients will generally be satisfied when I use home monitoring.
4.9 I expect that patients will generally cooperate with home monitoring.
4.10 I expect that I can count on support from direct colleagues when using home monitoring.
4.11 I expect that I can count on adequate support from Samen Digitaal.
4.12 My colleagues expect me to use home monitoring.
4.13 The management of the department expects me to use home monitoring.
4.14 Patients expect me to use home monitoring.
4.15 I expect to review the results of home monitoring in preparation for my consultation.
4.16 I expect to have enough knowledge to use home monitoring.
4.17 I am informed about the content of home monitoring.
4.18 I expect that home monitoring will reduce the number of follow-up consultations in this group.

4.19 I expect that some patients are not suitable for home monitoring.

4.20 I expect that home monitoring will cause uncertainty among patients.
4.21 I expect that, due to home monitoring, a recurrence will be detected later than in the current follow-up structure.
4.22 I expect that, with home monitoring, patients will need to come to the hospital less often.

4.23 I expect that, with home monitoring, care can be more focused on the patient.
4.24 I expect that patients with home monitoring will have a better quality of life than in regular follow-up.

**Organization**
5.1 I expect that protocols are available for the use of home monitoring.
5.2 I expect that there is enough staff to use home monitoring.
5.3 I expect that there is enough time available to use home monitoring in my daily work.
5.4 I expect that the patient has a computer/device available to use home monitoring.
5.5 I expect that there are enough people (Samen Digitaal, EvA Service Center) available to coordinate the implementation.
5.6 I expect that it is easy to find information about home monitoring in my organization.
5.7 I expect that there will be regular feedback on the progress of the implementation of home monitoring.

**Other Changes?**
6.1 Are there circumstances or changes in the department/hospital that influence the implementation of home monitoring?
Options:

- Yes
- No

6.1.1 If yes: Yes, namely:

**End**
7.1 Do you have any comments/additions?
(Open text field)

**____________________________________________________________________________**

**For patients – Example of the *‘Experienced Usability and Satisfaction with self-monitoring in the home Setting’* (GEMS) questionnaire**

**_____________________________________________________________________________**

Summary table presenting all questionnaire items (n=15).

Item Subscale Question or statement* Likert scale

1 Convenience of use It is frustrating to use 1-5

2 I had to learn a lot before I could use it properly. 1-5

3 Perceived value Home monitoring contribute to my health. 1-5

4 I think that it improves healthcare. 1-5

5 Efficiency of use home monitoring is easy to use. 1-5

6 I am losing too much time using it 1-5

7 Satisfaction How satisfied are you with it? 1-5

8 How likely is it that you would recommend it
to someone else who needs this care? 1-10

9 I would use it again. 1-10

10 How satisfied are you with the timing of the home
measurements? 1-5

11 How likely is it that you continue using it? Please elaborate. 1-5

Reference: Oudbier SJ, Smets EMA, Nieuwkerk PT, Neal DP, Nurmohamed SA, Meij HJ, et al. Patients' Experienced Usability and Satisfaction With Digital Health Solutions in a Home Setting: Instrument Validation Study. JMIR Med Inform. 2025;13:e63703. doi:10.2196/63703. Erratum in: JMIR Med Inform. 2025;13:e73416.
