# Supplementary material for: Towards personalised follow-up care in ovarian cancer using online remote PROMs monitoring: a study protocol of a feasibility trial
Source: BMJ Open. 2026 Mar 18;16(3):e113371. doi: 10.1136/bmjopen-2025-113371 (PMC13007198; doi:10.1136/bmjopen-2025-113371)
Supplement: Supplementary file 3 [file bmjopen-2025-113371.reviewer_comments.pdf]

PEER REVIEW HISTORY

BMJ Open publishes all reviews undertaken for accepted manuscripts. Reviewers are asked to complete a checklist review form and are provided with free text boxes to elaborate on their assessment. These free text comments are reproduced below.

ARTICLE DETAILS

Title (Provisional)

Towards personalized follow up care in ovarian cancer using online remote PROMs monitoring: a study protocol of a feasibility trial

Authors

Aarts, Johanna W.M.; Oudbier, S J; Van Muilekom, M M; Röling, P; Tromp, J; Smeulers, Marian; van Lonkhuijzen, Luc RCW

VERSION 1 - REVIEW

**Reviewer**	**1**
**Name**	**Kennedy, Fiona**
**Affiliation**	**University of Leeds, Psychosocial Oncology & Clinical Practice Research Group**
**Date**	**15-Dec-2025**
**COI**	**None**

Thank you for submitting this protocol. I have a number of comments/suggestions on this protocol. At present i do not think this protocol paper is sufficiently detailed to allow readers to know how the study was designed, what was carried out and to allow for replication. It's also unclear at what stage this study is at - for example has it started recruitment (if so provide dates) and/or completed etc.

Addressing the following points will strengthen the protocol manuscript and allow it to do what a protocol paper should do - e.g. providing explicit detail on the methods that otherwise may not be publicised, allowing for the prevention of unnecessary duplication and/or increases transparency, making it easier for others (editors, reviewers and readers) to see and understand any deviations from the protocol that occur during the conduct of the study.

1. Abstract: be more specific in the abstract methods on the home measurements being used. At one point you only mention 'weight'. I was also surprised to see no blood test monitoring (CA125) which is often a stand method used during follow-up of ovarian cancer in the UK.

2. What does MIDI stand for?

3. Make clear that the system is accessible for HCPs - this isn't clear in the abstract, although later in the paper it's clear that the results are integrated into the EHR or that's what i understood. Make this clear in the abstract too.

Strengths/Limitations summary points: point 2 - I didn't understand what was meant by 'and whether these change over time' - how is change assessed in the study?

Strengthen the statements in this section as use of words such as 'should' and 'may' are vague, and therefore clearer statements would be more beneficial.

Main paper:

1. Lines 5 and 11 - be more specific rather than phrases like 'a long time' or 'many years'.

2. Improve the context of how you are presenting the study - e.g. 'current follow-up care in OC is doctor-driven' - perhaps you should add 'in the Netherlands' or something to situate this statement. There is certainly a degree of nurse-led follow-up in the UK, although i'm not sure how widespread, so i don't think it's correct to say all follow-up care is doctor-driven. See also Kennedy et al papers below which illustrate how nurse-led follow-up is used.

3. No mention of CA125 blood test monitoring is a surprise. This seems to be standard practice for follow-up of ovarian cancer in the UK. As you state the limitations of physical examination, I'm surprised not to see some mention of blood test monitoring, or is that not used at all in Netherlands. Again, situating the study in the context is important here.

4. The statement about 'no studies' have explored this before is incorrect and should be changed. See Kennedy, F., Shearsmith, L., Holmes, M. et al. Electronic patient-reported monitoring of symptoms during follow-up of ovarian cancer patients: a feasibility study. BMC Cancer 22, 726 (2022). https://doi.org/10.1186/s12885-022-09817-5 and Kennedy F, Shearsmith L, Holmes M, Velikova G. 'It made me feel part of the team, having my homework to do' - women and specialist nurse experiences of remote follow-up after ovarian cancer treatment: a qualitative interview study. Support Care Cancer. 2022 Dec 13;31(1):2. doi: 10.1007/s00520-022-07470-z. PMID: 36512093; PMCID: PMC9745768.

5. Why 'weight' and 'waist circumference' chosen - no rationale given.

6. Are you providing weighing scales? You mention giving participants a tape measure.

7. Can patients access the system at other times or only at the reminded 3-monthly points? Make this clear.

8. Page 7 - unclear wording 'to find appropriate HCP nearby' - unclear what this section is saying - please reword to make explicit.

9. Description of measurement instruments is unclear (page 11), e.g.

- adapted version includes only the relevant questions (a protocol should allow for someone to know exactly what you did and replicate it in another study - this doesn't allow for this at all)

- what does GEMS stand for?

- how are all the measures/questionnaires rated/what are the response options? Given published validity/reliability data in OC context to back up why you are using these measures.

10. What is 'castor EDC' - it's not clear at all how this is used? Also related to data collection, it's unclear if the study outcomes are collected electronically or paper based?

11. How are you selecting patients for the focus groups? How do patients opt into this/ when are they invited and how? Purposive sampling employed at all? If so state and provide detail on what criteria you will try to sample - e.g. age, cancer stage

12. More explicit information and detail is required all all the aspects described briefly within the process evaluation section items - e.g. fidelity / compliance? What are you collecting to assess this and how?

13. What about withdrawal? Are you collecting how many patients ask to come off the study?

14. Unclear description of 'number of phone calls' 'visits' - 'done a year after intro of COPA'. Does this mean a year after each patient joins the study you will look at how many contacts they have had? What visits will be captured - all visits/contacts or only those specific to oncology?

15. What will happen if a study participant relapses? Is there call/visit data still collected? Will they be invited to give any further feedback or not?

16. Is any medical data (e.g. diagnosis / treatment) information collected and if so when, what and how? I would expect you'd need some of this to describe your sample at least.

17. Patient qualitative data - unclear. Interviews are mentioned in Box 1 but focus groups in main text. Be explicit and clear if doing both, and how many patients invited/included in either / how selected?

18. Sample size - i would expect to see reference to published guidance on the minimum number required for feasibility work.

19. Data Analysis Plan section - GEMS data analysis plan unclear (e.g. 'revised from 10-point to 5-point (Q8, Q9) - I don't follow what this means at all.

20. Qualitative interviews and/or focus groups - methods unclear and no detail on who is conducting these, where held, how sampled, and no detail provided on the analytic steps followed for thematic analysis. Just referencing Braun and Clarke isn't enough. Aspects of reflexivity should also be addressed.

21. Process evaluation data analysis section is poor.

22. Page 14 'The felt need...' - reword as unclear.

23. Why was ethics exempted? Give the name of the medical ethical review board or at least whether it was a hospital specific review board or other institution?

24. There are no dates provided as to whether this study has started, is mid-way through or completed recruitment? If ongoing, it would be useful to know how far in you are e.g. "To date XX patients have been recruited". Also, when is the data collected expected and when are the outcomes of the study expected providing estimated dates helps readers to know when to expect the study results.

25. Appendix I referenced in the main text is referred to as Appendix II in the actual appendix document.

VERSION 1 - AUTHOR RESPONSE

Reviewer: 1

Thank you for submitting this protocol. I have a number of comments/suggestions on this protocol. At present i do not think this protocol paper is sufficiently detailed to allow readers to know how the study was designed, what was carried out and to allow for replication. It's also unclear at what stage this study is at - for example has it started recruitment (if so provide dates) and/or completed etc.
Addressing the following points will strengthen the protocol manuscript and allow it to do what a protocol paper should do - e.g. providing explicit detail on the methods that otherwise may not be publicised, allowing for the prevention of unnecessary duplication and/or increases transparency, making it easier for others (editors, reviewers and readers) to see and understand any deviations from the protocol that occur during the conduct of the study.

1. Abstract: be more specific in the abstract methods on the home measurements being used. At one point you only mention 'weight'. I was also surprised to see no blood test monitoring (CA125) which is often a stand method used during follow-up of ovarian cancer in the UK.

We have added the full details of the home measurements in the abstract now. In the Netherlands blood test monitoring is not standard in follow up care, only when symptoms suspect a recurrence. This is also more common in other countries. We have not added this to the abstract, but instead have now added some sentences in the introduction to set the stage of the intervention and have added a more elaborate description of current follow up care in the Netherlands.

2. What does MIDI stand for?

We had stated this in the methods section, but not in the abstract. We have now added the full name in the abstract as well, i.e. Measurement Instrument for Determinants of Innovations (MIDI).

3. Make clear that the system is accessible for HCPs - this isn't clear in the abstract, although

later in the paper it's clear that the results are integrated into the EHR or that's what i understood. Make this clear in the abstract too.

We have now added this in the abstract.

Strengths/Limitations summary points: point 2 - I didn't understand what was meant by 'and whether these change over time' - how is change assessed in the study?
Strengthen the statements in this section as use of words such as 'should' and 'may' are vague, and therefore clearer statements would be more beneficial.

We have altered the strengths and limitation summary points, also taking the editor's notes into account.

Main paper:
1. Lines 5 and 11 - be more specific rather than phrases like 'a long time' or 'many years'.
We have specified this phrases now.

2. Improve the context of how you are presenting the study - e.g. 'current follow-up care in OC is doctor-driven' - perhaps you should add 'in the Netherlands' or something to situate this statement. There is certainly a degree of nurse-led follow-up in the UK, although i'm not sure how widespread, so i don't think it's correct to say all follow-up care is doctor-driven. See also Kennedy et al papers below which illustrate how nurse-led follow-up is used.

Thank you for addressing this. It is certainly the case that in some instances nurse-led follow up is present, but in most countries it is doctor-driven. We have now adjusted this sentence to make this nuance.

3. No mention of CA125 blood test monitoring is a surprise. This seems to be standard practice for follow-up of ovarian cancer in the UK. As you state the limitations of physical examination, I'm surprised not to see some mention of blood test monitoring, or is that not used at all in Netherlands. Again, situating the study in the context is important here.

There was a large RCT that showed that early detection of a asymptomatic recurrence through Ca-125 monitoring does not improve oncological outcomes. It even showed that starting treatment of an asymptomatic recurrence worsens quality of life of these patients. Therefore, in many countries, guidelines do not include standard CA-125 monitoring during follow up, such as those of the National Comprehensive Cancer Network in the USA (NCCN) or ESGO (European).
In the Netherlands patients receive 3-monthly visits at a gynaecologist or oncologist and does not include standard blood tests or imaging. We have added a few phrases in the introduction to address this.

4. The statement about 'no studies' have explored this before is incorrect and should be changed. See Kennedy, F., Shearsmith, L., Holmes, M. et al. Electronic patient-reported monitoring of symptoms during follow-up of ovarian cancer patients: a feasibility study. BMC Cancer 22, 726 (2022). https://doi.org/10.1186/s12885-022-09817-5 and Kennedy F, Shearsmith L, Holmes M, Velikova G. 'It made me feel part of the team, having my homework to do' - women and specialist nurse experiences of remote follow-up after ovarian cancer treatment: a qualitative interview study. Support Care Cancer. 2022 Dec 13;31(1):2. doi: 10.1007/s00520-022-07470-z. PMID: 36512093; PMCID: PMC9745768.

We regret that we have missed these very relevant and important studies. We have changed this sentence in the introduction.

5. Why 'weight' and 'waist circumference' chosen - no rationale given.
As we described we established a multidisciplinary stakeholder group to develop the intervention. In the group interviews/focus groups we organized with this group we discussed what home measurements, aside from PROMs, should be added. By consensus it was decided to add weight and waist circumference. Importantly, the patient representatives in the group were in favour of adding these two. Most women had experienced increase in waist circumference or weight change at time of diagnosis. They felt that adding these two measurements would increase the feeling of self-management and empowerment during follow up. We have added two phrases on the choice of these measurements.

6. Are you providing weighing scales? You mention giving participants a tape measure.
No we did not provide weighing scales. At inclusion the nurse asks if patients have a weighing scale. If not, it will be provided. For the weigh measurement, we are mainly interested in change of weight (increase or decrease) every three months. We therefore explain to patients that it is important to weigh themselves on the same scale every time.
We have added information on this in the methods section.

7. Can patients access the system at other times or only at the reminded 3-monthly points? Make this clear.
Yes, patients are able to access their patient record and this system at any time. They can send a message to the nurse. They can even fill out more questionnaires in the 3-month time frame if they need to. We have added this information.

8. Page 7 - unclear wording 'to find appropriate HCP nearby' - unclear what this section is saying - please reword to make explicit.
We regret that this section is unclear. We have rephrased it to clarify.

9. Description of measurement instruments is unclear (page 11), e.g.
- adapted version includes only the relevant questions (a protocol should allow for someone to know exactly what you did and replicate it in another study - this doesn't allow for this at all)
- what does GEMS stand for?
- how are all the measures/questionnaires rated/what are the response options? Given published validity/reliability data in OC context to back up why you are using these measures.

We regret that this section is unclear and that we did not provide sufficient information on the measurement instruments. We have added more information in the text. In addition, we have compiled a supplementary file including a detailed outline of all the measurements instruments and their items to support this section.

10. What is 'castor EDC' - it's not clear at all how this is used? Also related to data collection, it's unclear if the study outcomes are collected electronically or paper based?
We have added more information about this program that we use for data collection.

11. How are you selecting patients for the focus groups? How do patients opt into this/ when are they invited and how? Purposive sampling employed at all? If so state and provide detail on what criteria you will try to sample - e.g. age, cancer stage

One year after initiating the pilot study we will invite all patients who participate for at least 6 months in the program for a focus group. We will not make a specific selection. We aim at organizing two focus groups between 4 and 8 participants. We have added this information now in the methods section.

12. More explicit information and detail is required all the aspects described briefly within the process evaluation section items - e.g. fidelity / compliance? What are you collecting to assess this and how?
To provide more details about the process evaluation, we have added much more information on the several items of the process measures and how we define those in our specific study. We have also added a table including an overview of the process measures and how we collect those data.

13. What about withdrawal? Are you collecting how many patients ask to come off the study?
Yes, we also collect this information. We have now added that in the paper as well.

14. Unclear description of 'number of phone calls' 'visits' - 'done a year after intro of COPA'. Does this mean a year after each patient joins the study you will look at how many contacts they have had? What visits will be captured - all visits/contacts or only those specific to oncology?
We have specified this in the description of the process evaluation.

15. What will happen if a study participant relapses? Is there call/visit data still collected? Will they be invited to give any further feedback or not?
If a study participant relapses, they will no longer participate in a home monitoring system as they might start treatment which requires obviously a different type of organization of care. They will no longer receive the GEMS questionnaires. We have specified this in the paper now. However, we will still use the collected data of these patients for the evaluation of the intervention. With regard to the focus groups, we will ask the treating physician to decide whether it is appropriate to approach the patient to participate in the focus group.

16. Is any medical data (e.g. diagnosis / treatment) information collected and if so when, what and how? I would expect you'd need some of this to describe your sample at least.
We regret that we did not include this information. We have now added this.

17. Patient qualitative data - unclear. Interviews are mentioned in Box 1 but focus groups in main text. Be explicit and clear if doing both, and how many patients invited/included in either / how selected?
We have now specified this and made Box 1 and the main text consistent in wording.

18. Sample size - i would expect to see reference to published guidance on the minimum number required for feasibility work.
We have added references on which we based our sample size.

19. Data Analysis Plan section - GEMS data analysis plan unclear (e.g. 'revised from 10-point to 5-point (Q8, Q9) - I don't follow what this means at all.
We are sorry that we were not clear about this analysis plan. We have now elaborated on the data analysis of the GEMS questionnaire. Also, because we have added an example of the GEMS questionnaire in one of the appendices, we feel that this also supports understanding of this measurement instrument.

20. Qualitative interviews and/or focus groups - methods unclear and no detail on who is conducting these, where held, how sampled, and no detail provided on the analytic steps followed for thematic analysis. Just referencing Braun and Clarke isn't enough. Aspects of reflexivity should also be addressed.
We have added more information on this.

21. Process evaluation data analysis section is poor.
We have elaborated on this.

22. Page 14 'The felt need...' - reword as unclear.
We have rephrased the first part of this section.

23. Why was ethics exempted? Give the name of the medical ethical review board or at least whether it was a hospital specific review board or other institution?
The institutional review board of Amsterdam UMC evaluated the study protocol and decided that it was exempt from further review as this study was not subject to the Dutch Medical Research Involving Human Subject Act. It was a hospital specific review board. We added this.

24. There are no dates provided as to whether this study has started, is mid-way through or completed recruitment? If ongoing, it would be useful to know how far in you are e.g. "To date XX patients have been recruited". Also, when is the data collected expected and when are the outcomes of the study expected providing estimated dates helps readers to know when to expect the study results.
We started recruitment in January 2025 and up until now we have included 26 patients. We expect to reach our sample size of 50 next year.

25. Appendix I referenced in the main text is referred to as Appendix II in the actual appendix document.
We are sorry that we did not refer correctly to the Appendix. We have changed this.

VERSION 2 - REVIEW

**Reviewer**	**1**
**Name**	**Kennedy, Fiona**
**Affiliation**	**University of Leeds, Psychosocial Oncology & Clinical Practice Research Group**
**Date**	**09-Feb-2026**
**COI**	

Thank you for submitting the revision of this paper addressing the previous editor and reviewer comments. The manuscript is much improved in terms of the detail provided.

I do have some residual comments:

1. On page 5 you have added reference to the study previously exploring PROMs follow-up in ovarian cancer but you describe this as 2 studies - whereas in fact the papers are both drawing on one study but reporting different aspects of the study, so i think you should just refer to there being one other study and perhaps specify that this was conducted in the UK.

2. Ensure Figures are numbered correctly. I think Fig1 appears to be labelled as Fig2.

3. Use of colour in Figures - does the journal support this? Also in Table 1 - only LARS has orange indicating alerts being sent, should this be amended to show orange relating to SNAQ/weight item also, or it may make sense to remove the orange on LARS.

4. Page 11 - typo 'After 6(T1) en 12(T2)

5. Baseline participants demographics description in Box 1 - how is education levels measured? how is digital literacy and health literacy measured? A validated measure or something you have developed for the study? Are response options provided - if so it would be useful to know what options are.

6. Focus groups - when are they taking place? In Box suggests at 6m but in text it suggests at 1 year which is confusing. Perhaps remove the "One year after start of..." - as i think i understand that you would only include a participant in focus group who has been on the study for at least 6 months...?

7. Page 13 line 60 typo - 'focusgroups'

8. Page 15 line 32/33 - repeated sentence

9. Make clearer in the methods section who conducts the focus groups/interviews?

10. Reference of supplementary file II in the text suggests that it outlines measurement instruments but in reality it only outlines the MIDI and GEMS instruments, and not all instruments.

VERSION 2 - AUTHOR RESPONSE

<u>**Reviewer: 1**</u>
Comments to the Author:
Thank you for submitting the revision of this paper addressing the previous editor and reviewer comments. The manuscript is much improved in terms of the detail provided.

I do have some residual comments:
1. On page 5 you have added reference to the study previously exploring PROMs follow-up in ovarian cancer but you describe this as 2 studies - whereas in fact the papers are both drawing on one study but reporting different aspects of the study, so i think you should just refer to there being one other study and perhaps specify that this was conducted in the UK.

Reference 17 refers to the study performed in the UK, reference 18 refers to the PROMova study which was conducted in Denmark. So we do not believe that these references refer to the same study. We did add the country where these studies took place to be more detailed about the settings.

2. Ensure Figures are numbered correctly. I think Fig1 appears to be labelled as Fig2.
We are very sorry that this was not done correctly. We have checked it and should now be labelled as they should. Figure 1 refers to the figure with reported thresholds and figure 2 to the study design.

3. Use of colour in Figures - does the journal support this? Also in Table 1 - only LARS has orange indicating alerts being sent, should this be amended to show orange relating to SNAQ/weight item also, or it may make sense to remove the orange on LARS.

As the editor indicates, there is support for use of colour in figures. However, we agree with the reviewer that the orange colour does not contribute much in table 1 at the moment. Therefore, we have removed that orange shading.

4. Page 11 - typo 'After 6(T1) en 12(T2)
Thank you. We have changed this.

5. Baseline participants demographics description in Box 1 - how is education levels measured? how is digital literacy and health literacy measured? A validated measure or something you have developed for the study? Are response options provided - if so it would be useful to know what options are.

Thank you for the reviewer's eye on this detail, as this is indeed important to add. We have added the ways of assessing these in the footnote of Box 1.

6. Focus groups - when are they taking place? In Box suggests at 6m but in text it suggests at 1 year which is confusing. Perhaps remove the "One year after start of..." - as i think i understand that you would only include a participant in focus group who has been on the study for at least 6 months...?
Yes, that is what we meant. We regret this was unclear. We have changed this.

7. Page 13 line 60 typo - 'focusgroups'
We have corrected this.

8. Page 15 line 32/33 - repeated sentence
We have removed the part where we repeat ourselves.

9. Make clearer in the methods section who conducts the focus groups/interviews?
We have added this information on page 12.

10. Reference of supplementary file II in the text suggests that it outlines measurement instruments but in reality it only outlines the MIDI and GEMS instruments, and not all instruments.
The reviewer is right. We have corrected this.
